# “Flash” Solvent-free Synthesis of Triazoles Using a Supported Catalyst

**DOI:** 10.3390/molecules14010528

**Published:** 2009-01-22

**Authors:** Ibtissem Jlalia, Faouzi Meganem, Jean Herscovici, Christian Girard

**Affiliations:** 1 Laboratoire de Pharmacologie Chimique et Génétique UMR8151 CNRS - U640 INSERM – IFR2769, Ecole Nationale Supérieure de Chimie de Paris, 11, rue Pierre et Marie Curie, 75005 Paris, France; E-mails: ibtissemj@yahoo.fr (I. J.), jean-herscovici@enscp.fr (J. H.); 2 Laboratoire de Synthèse Organique et Application, Faculté des Sciences de Bizerte, Université du 7 Novembre à Carthage, 7021 Jarzouna Bizerte, Tunisia; E-mail: Faouzi.Meganem@fsb.rnu.tn (F. M.)

**Keywords:** Click chemistry, Huisgen’s cycloaddition, Copper (I) catalysis, Triazoles, Supported catalyst, Solvent-free.

## Abstract

A solvent-free synthesis of 1,4-disubstituted-1,2,3-triazoles using neat azides and alkynes and a copper(I) polymer supported catalyst (Amberlyst^®^ A21•CuI) is presented herein. As it provides the products in high yields and purities within minutes, this method thus being characterized as a "flash" synthesis, and was exemplified through the synthesis of a 24-compound library on a small scale.

## Introduction

Triazoles have gained in interest over the past few years following the introduction of the “click-chemistry” concept [1,2,3]. This approach concentrates on chemical reactions between highly reactive partners to provide ready access to structures that can be easily diversified, thanks to the generality of those reactions and their relative insensitivity to stereochemical and electronic considerations.

**Figure 1:**
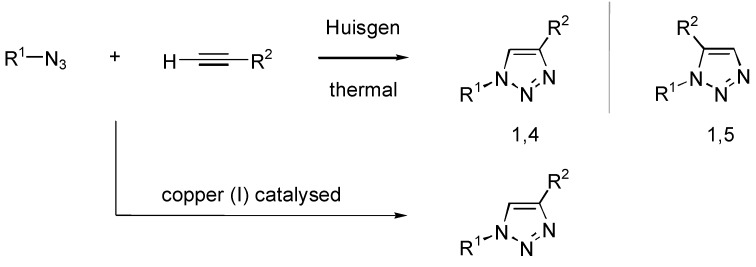
Huisgen’s cycloaddition route to 1,2,3-triazoles and its copper (I) catalyzed version.

Huisgen's thermal cycloaddition of azides and alkynes to give triazoles [4], was found to be catalyzed by copper(I) (Figure 1) [5,6,7,8,9,10]. These conditions illustrate the “click” concept perfectly. This facilitated the reaction at lower temperature and furthermore only the 1,4-disubstituted regioisomer of the 1,2,3-triazole was formed. The conditions used to conduct such reactions can be addition of copper (I) salts in organic or aqueous systems often in conjunction with a base [11,12,13], copper (II) salts/ ascorbic acid system (to generate the copper (I) species *in situ*) [14,15,16], copper salts adsorbed on zeolites [17], charcoal [18] or clay [19], copper wire [20,21,22] and nanoparticles/clusters [23,24].

We recently proposed a new catalytic system based on copper (I) iodide chelated on Amberlyst^®^ A21 resin, for use in automated solution synthesis of 1,2,3-triazoles from organic azides and terminal alkynes [25,26]. The advantages of this catalyst are the ease of preparation, a good catalytic activity and the simple separation from the reaction product by filtration. This catalyst was successfully employed for the synthesis of triazole libraries in solvents, the reaction needing however a few hours to overnight to be completed.

## Results and Discussion

**Scheme 1:**
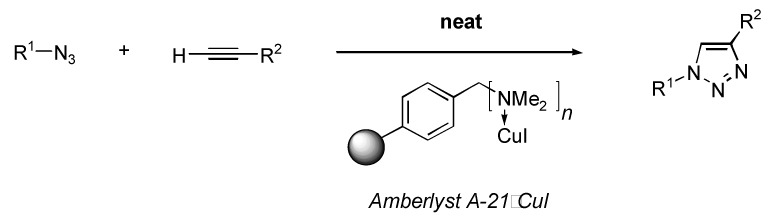
Solvent-free synthesis of triazoles using a polymer-supported copper (I) iodide.

In recent years there has been a growing pressure on organic chemists to not only find efficient reactions, that can achieve high yields and selectivities, but also to focus on the “greenness” of the processes [27]. One of the major environmental impacts of organic synthesis is the solvent use itself. During our work on this system, we found out that the A21•CuI polymer was able to catalyze triazole formation within only minutes using neat azides and alkynes derived from various organic structures, *i.e.* in a solvent-free manner (Scheme 1). 

We wish to report in this communication a new “flash” *(quasi instantaneous)* solvent-free method for the synthesis of triazoles using this polymer-supported catalyst [28,29,30,31]. When organic azides and terminal alkynes were mixed together and treated directly with A21•CuI catalyst, a rise in the mixture temperature was observed in most cases. As soon as the mixture cooled off, crystallization of the triazole usually occurred within five minutes and this was considered as the end of reaction (<0.5 h *vs.* more than 6 h in solution). Reaction products were then manually separated from the large beads of the catalyst. The results for the synthesis of 24 triazoles are presented in Table 1.

**Table 1:** Yields for the solvent-free synthesis of a series of triazoles^a^.

	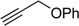	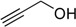	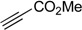	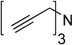	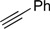	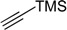
	2a	2b	2c	2d^[b]^	2e	2f^[c]^
PhCH_2_–N_3_	99	95	99	99	99	99
**1a**						
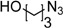	93	68	76	72	99	72
**1b**						
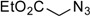	99	92	99	96	99	83
**1c**						
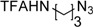	76	89	98	90	86	90
**1d**						

^a ^On a 0.5 mmol scale: 1 eq. alkyne/1.1 eq. azide, 30 mg (8 %mol) A21•CuI. All compounds gave correct mp, IR, ^1^H-, ^13^C-NMR and LC-MS analyses; ^b ^with 3.3 eq. azide; ^c ^0.75 eq. azide was used.

In most cases, the yields were high (average 90%) and for the two-thirds of the formed triazoles were between 90% and quantitative. In all reactions, only the 1,4-isomer was observed and all products were pure with some minor exceptions. As previously observed with this system, the small excess of azide used was not detected in the reaction products, suggesting a possible sequestration by the polymer.

Yields for the reactions of benzyl azide (**1a**), ethyl azidoacetate (**1c**) and 3-azidotrifluoro-acetamidopropane (**1d**) were good (averages between 89-98%). Products were usually isolated as highly crystalline solids and easily separated from the catalyst. Yields were lower, however, when using 3-azidopropanol (**1b**) and the corresponding triazoles were isolated in 80% average yield. This was mainly due to the sticky nature of the products which coated the polymer beads. In this case the reaction yields can be improved by washing the polymer beads with a solvent, but this step obviously diminishes the “greenness” of the approach.

The reaction yields of tripropargylamine (**2d**) were good when 3.3 eq. of the azide were used, and led exclusively to the corresponding tris(triazoles) **1a-d2d**, with no mono- or bis(triazoles) being observed. In this case, traces of copper leaked out from the catalyst due to the presence of the amine centre in the products. This is obviously one of the limitations of this system. However, residual minute amounts of copper can be quickly and easily removed from solutions of the products using polymer-supported thiourea [32].

Finally, trimethylsilylacetylene (**2f**) was found not to react as well as the other alkynes and leading to a coloration of the polymer beads (*light orange to red*) suggesting side reactions. In order to obtain better conversions, it was the only alkyne used in excess based onto the azide. Yields were also good here in most cases (average 86%), but still lower for the cycloaddition with **1b**.

## Conclusions

We have presented in this communication our findings concerning a new method for the synthesis of 1,4-disubstituted 1,2,3-triazoles. This method provides an “instantaneous” access to the products when using only neat alkynes, azides and an easily prepared polymer-supported copper (I) catalyst, thus being characterized as a “flash” reaction. This method is one of the fastest and easiest compared to traditional thermal and microwave-assisted procedures [33], or to the copper (I) catalyzed versions of Huisgen's cycloaddition themselves. The triazoles were obtained regioselectively in very good yields and purities in only a few minutes, thus improving the access to these heterocycles. We are convinced this method will find many applications for the synthesis of simple and more complex triazole containing molecules.

## Experimental

### General

*Chemicals*: Copper (I) iodide and propiolic acid methyl ester were purchased from Lancaster. Propargyl alcohol, tripropargylamine and trimethylsilylacetylene were purchased from Aldrich and phenylacetylene from Fluka. These chemicals were used without purification. Propargyl phenyl ether was prepared from propargyl bromide and phenol [34]. Azides were prepared from sodium azide and benzyl bromide, 3-chloropropanol, ethyl bromoacetate and 3-bromopropylamine hydrobromide (after treatment with ethyl trifluoroacetate) following published procedures [35,36,37,38]. *Solvents:* Acetonitrile (spectrometric grade, low water) was purchased from SDS France and used as such. Dichloromethane (SDS France) was treated with phosphorus pentaoxide at reflux (1 h) before being distilled. Melting points (mp) were determined using a Kofler apparatus after a first evaluation, calibration with a reference sample of a mp near the observed fusion and final measure of the melting point. Infrared spectra were recorded neat on a Jasco FT/IR-4100 in ATR mode (PIKE-MIRacle) between 4,000 and 400 cm^-1^ and are given in *ν* (cm^-1^). NMR spectra were recorded on a Bruker Avance DRX instrument in deuteriochloroform (unless otherwise noted) at 300 MHz for the ^1^H and 75.5 MHz for the ^13^C spectra. Chemical shifts (δ) are reported in part per million (ppm) relative to the tetramethylsilane signal as an internal reference. Couplings constants (*J*) are in hertz and signal multiplicities indicated as s (singlet), d (doublet), t (triplet), q (quadruplet), m (multiplet), dd (doublet of doublets). LC-MS analyses were done on a Shimadzu LCSM-2010 A instrument equipped with a SPD-M10 A PDA diode array detector (D_2_, lamp from 190 to 400 nm) and an ELSD-LT light scattering detector on an Alltima HP C8 3m (Alltech) reversed phase (L= 53 mm; ID = 7 mm) HPLC column. The LC were ran using a 1 mL/min flow using a gradient between acetonitrile and water containing formic acid (0,1%): 0 to 1 min: 30% CH_3_CN, 1 to 5 min: from 30% to 100% CH_3_CN, 5 to 12 min : 100% CH_3_CN, 12 to 14,99 min: from 100% to 30% CH_3_CN, 14,99 to 20 min: 30% CH_3_CN. MS was recorded between *m/z* = 100 to 500 at the exit of the column using an ESI ionization and positive ion mode (detector= 1.5 kV, quadripole = 5 V).

### Procedures

*Dry Amberlyst^®^ A-21:* Commercial wet Amberlyst *^®^* A21 resin (Aldrich, 20-50 mesh, 100 g) was suspended in MeOH (500 mL) for 0.5 h and filtered (3 times) and then soaked in methylene chloride (500 mL) for 0.5 h and again filtered (3 times). The resulting resin was placed in a round-bottom flask on a rotoevaporator and dried at 50 °C under 10 mm Hg until it was free-flowing. The dried resin was then kept overnight under vacuum in a desiccator over P_2_O_5_. Specifications from the manufacturer indicate that the polymer contains 4.8 meq. of amine/g of dry resin.

*Preparation of the supported catalyst (A-21.CuI):* Dry Amberlyst *^®^* A21 (1.0 g, 4.8 mmol amine) was added to a solution of copper (I) iodide (381 mg, 2.00 mmol) in acetonitrile (15 ml) and gently shaken on an orbital stirrer for 17 h. The solvent was drawn off and the resin washed with CH_3_CN (2x15 mL), CH_2_Cl_2_ (2x15 mL) and dried under vacuum (0.01 mm Hg) at 40°C. The weight increase was 0.307 g (1.61 mmol CuI), which gave a polymer loading of 1.23 mmol CuI∙g^-1^. Elemental analyses (Service Central d'Analyses du CNRS, Solaize, France) gave a copper content of 8.64 %, indicative of a loading of 1.35 mmol CuI∙g^-1^.

### General procedure for triazole synthesis

**CAUTION! ***Organic azides are potentially explosive and should be handle with care. Even if no incident occurred in this solvent-free reaction on this scale, the cycloaddition can be highly exothermic and should not be attempted on a larger scale, without being aware of explosion risks.*

The azide (0,55 mmol) and alkyne (0,50 mmol) were placed together in an open small test tube. Amberlyst *^®^* A21•CuI (1.35 mmol/g, 30 mg, 0.040 mmol, 8% mol) was added at once. A quick temperature rise was observed in most cases and the triazole crystallized out generally within 5 minutes. After half an hour, which was selected arbitrarily, the product was separated from the catalyst either manually or by dissolution in CH_2_Cl_2_ or CH_3_CN (3 x 1 mL) and recovered after evaporation of the solvent.

*1-Benzyl-4-(phenoxymethyl)triazole* (**1a2a**): Prepared from 66 mg (0.50 mmol) propargyl phenyl ether and 73 mg (0.55 mmol) benzyl azide . The product was obtained as a white solid (131 mg, 99 %). C_16_H_15_N_3_O, M = 265.31 g∙mol^-1^; mp: 119-121 °C; FTIR: *ν* 3132, 3016, 2970, 2920, 2866, 1588, 1488, 1239, 1222 and 1052 cm^-1^; ^1^H-NMR: *δ* 5.17 (s, 2H), 5.51 (s, 2H), 6.95 (m, 3H), 7.26 (m, 4H), 7.33 (m,3H) and 7.44 (s, 1H) ppm; ^13^C-NMR: *δ* 54.2, 62.0, 114.8, 121.3, 128.1, 128.8 , 129.1, 129.5, 134.5, 144.6 and 158.2 ppm; LC-MS: ELSD pur. 92 %, UV pur. 100 %; R_t _= 9.56 min; *m/z*: 266 ([M+H]^+^).

*3-(4-Phenoxytriazol-1-yl)propan-1-ol* (**1b2a**): Prepared from 66 mg (0.50 mmol) propargyl phenyl ether and 56 mg (0.55 mmol) 3-azidopropanol. The product was obtained as an off-white solid (108 mg, 93 %). C_12_H_15_N_3_O_2_, M = 233.27 g∙mol^-1^; mp: 39-41°C; FTIR: *ν* 3304, 3132, 3107, 2945, 2870, 1600, 1488, 1239, 1218 and 1052 cm^-1^;^ 1^H-NMR: *δ* 2.10 (q, *J=*6.0 Hz, 2H), 3.23 (s, 1H), 3.61 (t, *J=*6.0 Hz, 2H), 4.49 (t, *J=*6.0 Hz, 2H), 5.16 (s, 2H), 6.96 (m, 3H), 7.27 (m, 2H) and 7.66 (s, 1H) ppm;^ 13^C-NMR: *δ* 32.6, 47.1, 58.5, 61.9, 114.7, 121.3, 123.2, 129.5, 144.1 and 158.2 ppm; LC-MS: ELSD pur. 99 %, UV pur. 100 %; R_t _= 4.08 min; *m/z*: 234 ([M+H]^+^).

*Ethyl 2-[4-(phenoxymethyl)triazol-1-yl]acetate* (**1c2a**): Prepared from 66 mg (0.50 mmol) propargyl phenyl ether and 71 mg (0.55 mmol) ethyl azidoacetate. The product was obtained as a pale yellow oily solid (129 mg, 99 %). C_13_H_15_N_3_O_3_, M = 261.28 g∙mol^-1^; FTIR: *ν* 3153, 2944, 2962, 2879, 1746, 1596, 1483, 1471, 1401, 1235 , 1210, 1177 and 1031 cm^-1^; ^1^H-NMR: *δ* 1.27 (t, *J*=7.2 Hz, 3H), 4.26 (q, *J*=7.2 Hz, 2H), 5.12 (s, 2H), 5.20 (s, 2H, H-7), 6.95 (m, 3H), 7.27 (m,2H) and 7.73 (s, 1H) ppm; ^13^C-NMR: *δ* 14.0, 50.8, 61.8, 62.4, 114.8, 121.2, 124.3, 129.5, 144.5, 158.2 and 166.2 ppm; LC-MS : ELSD pur. 97 %, UV pur. 100 %; R_t _= 8.66 min; *m/z*: 262 ([M+H]^+^).

*2,2,2-Trifluoro-N-[3-(4-phenoxymethyl-[1,2,3]triazol-1-yl)-propyl]-acetamide* (**1d2a**): Prepared from 66 mg (0.50 mmol) propargyl phenyl ether and 108 mg (0.55 mmol) *N*-(trifluoracetyl)-1-azido-3-aminopropane. The product was obtained as a white solid (126 mg, 76 %). C_14_H_15_F_3_N_4_O_2_, M= 328.30 g∙mol^-1^. mp: 78-80°C; FTIR: *ν* 3356, 3140, 3102, 2958, 2883, 1704, 1600, 1559, 1488, 1243, 1206 and 1168 cm^-1^; ^1^H-NMR (CD_3_CN): *δ* 2.16 (q, *J=*6.92 Hz, 2H), 3.32 (t, *J=*6.5 Hz, 2H), 4.42 (t, *J=*6.5 Hz, 2H), 5.17 (s, 2H), 6.97 (m, 3H) 7.28 (m, 2H), 7.80 (s, 1H) and 7.90 (s,1H) ppm;^ 13^C-NMR (CD_3_CN): *δ* 29.1, 37.0, 47.7, 61.7, 114.7, 117.7, 121.4, 123.3, 129.6, 144.5, 157.6 and 158.1 ppm; LC-MS : ELSD pur. 99 %, UV pur. 100 %; R_t _= 8.37 min; *m/z*: 329 ([M+H]^+^).

*(1-Benzyltriazol-4-yl)methanol* (**1a2b**): Prepared from 28 mg (0.50 mmol) propargyl alcohol and 73 mg (0.55 mmol) benzyl azide. The product was obtained as white solid (89 mg, 95 %). C_10_H_11_N_3_O, M = 189.22 g∙mol^-1^; mp: 76-78°C; FTIR: *ν* 3257, 3144, 3091, 2953, 2920, 1451, and 1048 cm^-1^; ^1^H-NMR: *δ* 4.11 (s, 1H), 4.78 (s, 2H), 5.46 (s, 2H), 7.30 (m, 5 H) and 7.91 (s, 1H) ppm; ^13^C-NMR: *δ* 54.1, 56.0, 122.0, 128.1, 128.7 , 129.1, 134.5 and 148.0 ppm; LC-MS : ELSD pur. 97%, UV pur. 100%; R_t _= 3.41 min; *m/z*: 190 ([M+H]^+^).

*3-[4-(Hydroxymethyl)triazol-1-yl]propan-1-ol* (**1b2b**): Prepared from 28 mg (0.50 mmol) propargyl alcohol and 56 mg (0.55 mmol) 3-azidopropanol. The product was obtained as a viscous colorless oil (53 mg, 68%). C_6_H_11_N_3_O_2_, M = 157.17 g∙mol^-1^; FTIR: *ν* 3382, 3142, 2944, 2881, 1658, 1437, 1344, 1219, 1138 and 1056 cm^-1^;^ 1^H-NMR (CD_3_CN) : *δ* 2.02 (q, *J=*6.0 Hz, 2H), 3.24 (s, 1H), 3.49 (t, *J=*6.0 Hz, 2H), 4.41 (t, *J=*6.0 Hz, 2H), 4.63 (s, 2H) and 7.66 ( s, 1H) ppm; ^13^C-NMR (CD_3_CN): *δ* 33.8, 47.5, 56.5, 58.8, 122.8 and 148.9 ppm; LC-MS: ELSD pur. 90 %, UV pur. 100 %; R_t _= 2.67 min ; *m/z*: 158 ([M+H]^+^).

*Ethyl 2-[4-(hydroxymethyl)triazol-1-yl]acetate* (**1c2b**): Prepared from 28 mg (0.50 mmol) propargyl alcohol and 71 mg (0.55 mmol) ethyl azidoacetate. The product was obtained as a pale yellow oily solid (95 mg, 92 %). C_7_H_11_N_3_O_3_, M = 185.18 g∙mol^-1^; FTIR: *ν* 3110, 3076, 3038, 2849, 1708, 1210 and 1023 cm^-1^; ^1^H-NMR: d 1.26 (t, *J*=7.2 Hz, 3H), 4.21 (q, *J*=7.2 Hz, 2H), 4.72 (s, 2H), 5.12 (s, 2H) and 7.67 (s, 1H) ppm; ^13^C-NMR: *δ* 14.0, 50.8, 56.1, 62.4, 123.8, 148.3 and 166.5 ppm; LC-MS: ELSD pur. 99 %, UV pur. 100%; R_t _= 3.02 min; *m/z*: 186 ([M+H]^+^), 208 ([M+Na])^+^.

*2,2,2-Trifluoro-N-[3-(4-hydroxymethyl-[1,2,3]triazol-1-yl)-propyl]-acetamide* (**1d2b**): Prepared from 28 mg (0.50 mmol) propargyl alcohol and 108 mg (0.55 mmol) *N*-(trifluoracetyl)-1-azido-3-amino-propane. The product was obtained as a beige solid (112 mg, 89 %). C_8_H_11_F_3_N_4_O_2_. M= 252.20 g∙mol^-1^;mp: 90-92°C; FTIR: *ν* 3306, 3219, 3061, 2949, 1721, 1576, 1467, 1181 and 1069 cm^-1^; ^1^H-NMR (CD_3_CN): *δ* 2.16 (q, *J=*6.5 Hz, 2H), 3.40 (t, *J=*6.5 Hz, 2H), 4.41 (t, *J=*6.0 Hz, 2H), 4.65 (s, 2H), 7.78 (s, 1H) and 7.90 (s, 1H) ppm;^ 13^C-NMR (CD_3_CN): *δ* 28.9, 36.6, 38.6, 47.9, 55.0 and 122.7 ppm; LC-MS: ELSD pur. 99 %, UV pur. 100 %; R_t _= 2.07 min; *m/z*: 253 ([M+H]^+^).

*Methyl 1-benzyltriazole-4-carboxylate* (**1a2c**): Prepared from 42 mg (0.50 mmol) propiolic acid methyl ester and 73 mg (0.55 mmol) benzyl azide . The product was obtained as an off-white solid (108 mg, 99 %). C_11_H_11_N_3_O_2_, M = 217.23 g∙mol^-1^; mp: 115-117 °C; FTIR: *ν* 3112, 3066, 3038, 2849, 1725, 1538, 1239 and 1048 cm^-1^; ^1^H-NMR: *δ* 3.90 (s, 3H), 5.55 (s, 2H), 7.30 (m, 2H), 7.38 (m, 3H) and 8.02 (s, 1H) ppm; ^13^C-NMR: *δ* 52.2, 54.5, 127.3, 128.3, 129.2, 129.3, 133.6, 140.3 and 161.1 ppm; LC-MS: ELSD pur. 98 %, UV pur. 100 %; R_t _= 8.88 min; *m/z*: 218 ([M+H]^+^).

*Methyl 1-(3-hydroxypropyl)triazole-4-carboxylate* (**1b2c**): Prepared from 42 mg (0.50 mmol) propiolic acid methyl ester and 56 mg (0.55 mmol) 3-azidopropanol. The product was obtained as a beige solid (70 mg, 76 %). C_7_H_11_N_3_O_3_, M = 185.18 g∙mol^-1^; mp: 55-57 °C; FTIR: *ν* 3124, 2959, 2875, 1737, 1721, 1543, 1223 and 1044 cm^-1^; ^1^H-NMR: *δ* 2.18 (q, *J=*6.0 Hz, 2H), 3.67 (t, *J=*6.0 Hz, 2H), 3.93 (s, 3H), 4.62 (t, *J=*6.0 Hz, 2H) and 8.22 (s, 1H) ppm; ^13^C-NMR: *δ* 32.4, 47.5, 52.2, 58.3, 128.2, 139.7 and 161.2 ppm; LC-MS: ELSD pur. 96 %, UV pur. 100 %; R_t _= 2.98 min; *m/z*: 186 ([M+H]^+^), 208 ([M+Na])^+^.

*Methyl 1-(ethoxycarbonylmethyl)triazole-4-carboxylate* (**1c2c**): Prepared from 42 mg (0.50 mmol) propiolic acid methyl ester and 71 mg (0.55 mmol) ethyl azidoacetate. The product was obtained as a beige solid (105 mg, 99 %). C_8_H_11_N_3_O_4_, M = 213.19 g∙mol^-1^; mp: 102-104°C; FTIR: *ν* 3149, 3007, 2968, 1765, 1718, 1543, 1443, 1380, 1219 and 1032 cm^-1^; ^1^H-NMR: *δ* 1.27 (t, *J*=7.2 Hz, 3H), 3.92 (s, 3H), 4.25 (q, *J*=7.2 Hz, 2H), 5.22 (s, 2H) and 8.27 (s, 1H) ppm;^ 13^C-NMR: *δ* 14.2, 50.8, 52.4, 62.8 and 129.3 ppm; LC-MS: ELSD pur. 91 %, UV pur. 100 %; R_t _= 3.68 min; *m/z*: 214 ([M+H]^+^).

*1-[3-(2,2,2-Trifluoro-acetylamino)-propyl]-1H-[1,2,3]triazole-4-carboxylic acid methyl ester* (**1d2c**): Prepared from 42 mg (0.50 mmol) propiolic acid methyl ester and 108 mg (0.55 mmol) *N*-(trifluoracetyl)-1-azido-3-aminopropane. The product was obtained as a beige solid (138 mg, 98 %). C_9_H_11_F_3_N_4_O_3_, M= 280.21 g∙mol^-1^; mp: 66-68°C; FTIR: *ν* 3290, 3137, 3094, 2962, 1559, 1219, 1166 and 1048 cm^-1^; ^1^H-NMR (CD_3_CN): *δ* 2.18 (q, *J=*6.0 Hz, 2H), 3.37 (t, *J=*6.0 Hz, 2H), 4.45 (t, *J=*6.0 Hz, 2H), 4.50 (t, *J=*6.5, 2H), 7.86 (s, 1H) and 8.35 (s, 1H) ppm;^ 13^C-NMR (CD_3_CN): *δ* 28.5, 37.6, 49.1, 50.5, 126.2, 128.1 , 133.9 and 161.1 ppm; LC-MS: ELSD pur. 80 %, UV pur. 100 %; R_t _= 3.32 min; *m/z*: 281 ([M+H]^+^).

*Tris-(4-benzyl-[1,2,3]triazol-1-ylmethyl)-amine* (**1a2d**): Prepared from 69 mg (0.50 mmol) tripropargylamine and 220 mg (1.65 mmol) benzyl azide. The product was obtained as a white solid (262 mg, 99 %). C_30_H_30_N_10_, M = 530.64 g∙mol^-1^; mp: 146-148 °C; FTIR: *ν* 3139, 3098, 3062, 2950, 2934, 1634, 1429, 1332, 1091 and 1050 cm^-1^; ^1^H-NMR: *δ* 3.69 (s, 6H), 5.50 (s, 6H), 7.26 (m, 6H), 7.34 (m, 9H) and 7.66 (s, 3H) ppm; ^13^C-NMR: *δ* 47.3, 54.2, 123.8, 128.1, 128.7, 134.9 and 144.4 ppm; LC-MS: ELSD pur. 96 %, UV pur. 100 %; R_t _= 6.5 min; *m/z*: 531 ([M+H]^+^).

*Tris(4-(3-hydroxy-propoyl)-[1,2,3]triazol-1-ylmethyl)amine* (**1b2d**): Prepared from 69 mg (0.50 mmol) tripropargylamine and 167 mg (1.65 mmol) 3-azidopropanol. The product was obtained as pale green solid (156 mg, 72 %). C_18_H_30_N_10_O_3_, M = 434.50 g∙ mol^-1^; mp: 104-106 °C; FTIR: *ν* 3400, 3139, 2950, 2919, 1644, 1460, 1329 and 1055 cm^-1^; ^1^H-NMR: *δ* 1.57 (m, 6H), 3.70 (s, 6H), 4.74 (s,3H), 5.16 (t, *J=4.5*, 6H), 5.30 (m, 6H) and 7.87 (s, 3H) ppm; ^13^C-NMR: *δ* 31.3, 45.9, 48.3, 56.8 and 123.3 ppm; LC-MS: ELSD pur. 90 %, UV pur. 100%; R_t _= 2 min; *m/z*: 436 ([M+H]^+^).

*Tris((4-ethoxycarbonylmethyl)-[1,2,3]triazol-1-ylmethyl)amine* (**1c2d**): Prepared from 69 mg (0.50 mmol) tripropargylamine and 213 mg (1.65 mmol) ethyl azidoacetate. The product was obtained as a beige solid (249 mg, 96 %). C_21_H_30_N_10_O_6_, M = 518.53 g∙mol^-1^; mp: 110-112 °C; FTIR: *ν* 3144, 2986, 2939, 1735, 1639, 1224 and 1045 cm^-1^; ^1^H-NMR: *δ* 1.29 (t, *J=6.9*, 9H), 3.83 (s, 6H), 4.25 (q, *J=7.2*, 3H), 5.50 (s, 6H) and 7.87 (s, 3H) ppm; ^13^C-NMR: *δ* 14.5, 51.0, 62.3, 110.0 and 166.3 ppm; LC-MS: ELSD pur. 90 %, UV pur. 100%; R_t _= 3.2 min; *m/z*: 519 ([M+H]^+^).

*Tris[4-(3-(2,2,2,-trifluoro-acetylamino)-propyl)]-[1,2,3]triazol-1-ylmethyl)amine* (**1d2d**): Prepared from 78 mg (0.50 mmol) tripropargylamine and 108 mg (1.65 mmol) *N*-(trifluoracetyl)-1-azido-3-aminopropane. The product was obtained as a beige oily solid (324 mg, 90 %). C_21_H_30_F_9_N_13_O_3_, M = 719.58 g∙mol^-1^; FTIR: *ν* 3269, 3133, 3087, 2939, 2897, 1716, 1565, 1445, 1355, 1215, 1203, 1150 and 1049 cm^-1^; ^1^H-NMR (CD_3_CN): *δ* 2.16 (q, *J=*6.5 Hz, 2H), 3.27 (t, *J=*6.5 Hz, 2H), 3.71 (s, 6H), 4.37 (t, *J=*6.0 Hz, 2H), 7.78 (s, 3H) and 7.90 (s, 3H) ppm; ^13^C-NMR: *δ* 29.0, 36.8, 47.2, 47.6 144.3 and 157.8 ppm; LC-MS: ELSD pur. 92 %, UV pur. 90 %; R_t _= 1.86 min; *m/z*: 720 ([M+H]^+^).

*1-Benzyl-4-phenyl-1H-[1,2,3]triazole* (**1a2e**): Prepared from 51 mg (0.50 mmol) phenylacetylene and 73 mg (0.55 mmol) benzyl azide. The product was obtained as a white solid (117 mg, 99 %). C_15_H_13_N_3_, M = 235.29 g∙mol^-1^; mp: 126-128 °C; FTIR: *ν* 3144, 3037, 2975, 1496 and 1044 cm^-1^; ^1^H-NMR: *δ* 5.53 (s, 2H), 7.32 (m, 8H), 7.65 (s, 1H) and 7.77 (m, 2H) ppm; ^13^C-NMR: *δ* 54.2, 54.5, 127.3, 128.3, 129.2, 129.3, 134.5, 133.6, 140.2 and 161.1 ppm; LC-MS: ELSD pur. 93 %, UV pur. 100 %; R_t _= 8.88 min; *m/z*: 236 ([M+H]^+^).

*3-(4-Phenyl-[1,2,3]triazol-1-yl)-propan-1-ol* (**1b2e**): Prepared from 51 mg (0.50 mmol) phenylacetylene and 56 mg (0.55 mmol) 3-azidopropanol. The product was obtained as a white solid (102 mg, 99 %). C_12_H_15_N_3_O_2_, M = 203.25 g∙mol^-1^; mp: 90-92°C; FTIR: *ν* 3315, 3120, 2950, 2875, 1600 and 1052 cm^-1^;^ 1^H-NMR: *δ* 2.18 (q, *J=*6.0 Hz, 2H), 3.67 (t, *J=*6.0 Hz, 2H), 3.93(s, 1H), 4.62 (t, *J=*6.0 Hz, 2H), 7.59 (m, 3H), 7.83 (m, 2H) and 7.84 (s, 1H);^ 13^C-NMR: *δ* 32.4, 47.5, 52.2, 58.3, 128.2, 139.7 and 161.2 ppm; LC-MS: ELSD pur. 98 %, UV pur. 100 %; R_t _= 2.96 min; *m/z*: 204 ([M+H]^+^).

*(4-Phenyl-[1,2,3]triazol-1-yl)-acetic acid ethyl ester* (**1c2e**): Prepared from 51 mg (0.50 mmol) phenylacetylene and 71 mg (0.55 mmol) ethyl azidoacetate. The product was obtained as a white solid (114 mg, 99 %). C_12_H_13_N_3_O_2_, M = 231.26 g∙mol^-1^; mp: 102-104°C; FTIR: *ν* 3140, 3079, 3004, 2950, 1758, 1464, 1082 and 1044 cm^-1^; ^1^H-NMR: *δ* 1.27 (t, *J*=7.2 Hz, 3H), 4.23 (q, *J*=7.2 Hz, 2H), 5.13 (s, 2H), , 7.35 (m,3H), 7.79 (m,2H) and 7.89 (s, 1H);^13^C-NMR: *δ* 14.4, 51.4, 52.7, 63.2, 129.4, 161.4 and 166.0 ppm; LC-MS: ELSD pur. 99 %, UV pur. 100 %; R_t _= 7.86 min; *m/z*: 232 ([M+H]^+^).

*2,2,2-Trifluoro-N-[3-(4-phenyl-[1,2,3]triazol-1-yl)-propyl]-acetamide* (1d2e): Prepared from 51 mg (0.50 mmol) phenylacetylene and 108 mg (0.55 mmol) *N*-(trifluoracetyl)-1-azido-3-aminopropane. The product was obtained as a white solid (128 mg, 86 %). C_13_H_13_F_3_N_4_O, M= 298.27 g∙mol^-1^; mp: 158-160°C; FTIR: *ν* 3211, 3045, 2953, 2896, 1721, 1193, and 1144 cm^-1^; ^1^H-NMR: *δ* 2.25 (m, 2H), 3.43(t, *J=*6.5 Hz, 2H), 4.50 (t, *J=*6.5 Hz, 2H), 7.05 (s, 1H), 7.39 (m, 4H) and 7.82 (m, 2H) ppm;^ 13^C-NMR: *δ* 28.7, 36.6, 47.2, 117.7, 121.4, 125.1, 127.8, 128.8, 130.7, 146.2, 157.6 and 158.1 ppm; LC-MS: ELSD pur. 96 %, UV pur. 100 %; R_t _= 7.88 min; *m/z*: 299 ([M+H]^+^).

*1-Benzyl-4-trimethylsilanyl-1H-[1,2,3]-triazole* (1a2f): Prepared from 49 mg (0.50 mmol) (trimethylsilyl)acetylene and 50 mg (0.375 mmol) benzyl azide. The product was obtained as a pale green solid (86 mg, 99 %). C_12_H_17_N_3_Si, M = 231.38 g∙mol^-1^; mp: 74-76°C; FTIR: *ν* 3286, 3115, 3061, 2953, 2920, 1674, 1542, 1438, 1318, 1280 and 1168 cm^-1^; ^1^H-NMR: *δ* 0.11 (s, 9H), 5.56 (s, 2H), 7.24-7.34 (m, 5 H), 7.26 (s, 1H) ppm; ^13^C-NMR: *δ* 0.02, 54.6, 129.2, 129.4, 130.2 and 136.1 ppm; LC-MS: ELSD pur. 96%, UV pur. 100%; R_t _= 11.23 min; *m/z*: 232 ([M+H]^+^).

*3-(4-(trimethylsilyl)-1H-1,2,3triazol-1-yl)propan-1-ol* (**1b2f**): Prepared from 49 mg (0.50 mmol) (trimethylsilyl)acetylene and 38 mg (0.375 mmol) 3-azidopropanol. The product was obtained as a pale green solid (54 mg, 72%). C_8_H_17_N_3_OSi, M = 199.33 g∙mol^-1^; mp :58-60°C; FTIR: *ν* 3290, 2920, 2850, 1650, 1461, 1213,1172 and 1049 cm^-1^;^ 1^H-NMR : *δ* 0.12 (s,9H), 1.94 (q, *J*=2Hz, 2H), 3.44-3.47 (m, 2H), 3.55-3.58 (t, *J*=4.5Hz, 1H), 4.34-4.38 (t, *J*=6Hz, 2H) and 7.41 (s, 1H) ppm; ^13^C-NMR: *δ* 0.02, 34.1, 47.6, 59.7 and 130.7 ppm; LC-MS: ELSD pur. 90 %, UV pur. 100 %; R_t _= 2.74 min ; *m/z*: 200 ([M+H]^+^).

*Ethyl 2-(4-(trimethylsilyl)-1H-1,2,3-triazol-1-yl)acetate* (1c2f): Prepared from 49 mg (0.50 mmol) (trimethylsilyl)acetylene and 48 mg (0.375 mmol) ethyl azidoacetate. The product was obtained as a brown oil (70 mg, 83 %). C_9_H_17_N_3_O_2_Si, M = 227.34 g∙mol^-1^; FTIR: *ν* 2928, 2854, 1746, 1455, 1372, 1213 and 1023 cm^-1^; ^1^H-NMR: d 0.12 (s, 9H), 1.12 (t, *J*=8Hz, 3H), 4.07-4.09 (q, *J*=1 Hz, 2H), 5.01(s, 1H) and 7.49 (s, 1H) ppm; ^13^C-NMR: *δ* 0.02, 15.2, 51.4, 63.5, 131.5 and 167.7 ppm; LC-MS: ELSD pur. 99 %, UV pur. 100%; R_t _= 8.50 min; *m/z*: 228 ([M+H]^+^).

*2,2,2-trifluoro-N-(3-(4-trimethylsilyl)-1H-1,2,3triazole-1-yl)propyl)acetamide* (**1d2f**): Prepared from 46 mg (0.50 mmol) (trimethylsilyl)acetylene and 59 mg (0.375 mmol) *N*-(trifluoracetyl)-1-azido-3-aminopropane. The product was obtained as a pale green solid (101 mg, 90 %). C_10_H_17_F_3_N_4_OSi, M = 294.35 g∙mol^-1^; mp: 120-122°C; FTIR: *ν* 3186, 3124, 3073, 2962, 1721, 1571, 1185 and 1156 cm^-1^; ^1^H-NMR (CD_3_CN) : δ 0.15 (s, 9H), 1.82 (t, *J*=3Hz, 2H), 3.15-3.17 (m, 2H), 4.26-4.30 (q, *J*=3Hz, 2H), 7.49-7.50 (m, 1H) and 7.68 (s, 1H) ppm;^ 13^C-NMR (CD_3_CN): *δ* 0.02, 28.7, 36.5, 47.7, 132.6 and 157.7 ppm; LC-MS: ELSD pur. 99 %, UV pur. 100%; R_t _= 10.15 min; *m/z*: 335 ([M+MeCN]^+^).
